# Neo-antigen specific cancer vaccines for acute lymphoblastic leukemia—challenges, opportunities, and future directions

**DOI:** 10.1007/s00262-025-04107-y

**Published:** 2025-06-23

**Authors:** Victoria Bloch Blytt Sandstad, Signe Modvig, Morten Orebo Holmström

**Affiliations:** 1 Department of Clinical Immunology, Copenhagen University Hospital Rigshospitalet, Copenhagen, Denmark; 2 Department of Clinical Medicine, Copenhagen University, Copenhagen, Denmark; 3 National Center for Cancer Immune Therapy, Department of Oncology, Copenhagen University Hospital Herlev-Gentofte, Borgmester Ib Juuls Vej 9 4th floor, DK-2730 Herlev, Denmark

**Keywords:** Acute lymphoblastic leukemia, Neo-antigens, Cancer vaccines, Personalized vaccines, Immune escape

## Abstract

Acute lymphoblastic leukemia (ALL) is the most common pediatric malignancy, with standard treatment consisting of intensive chemotherapy and corticosteroids. While curative in most cases, this regimen leads to significant toxicity and long-term sequelae. Recent advancements in cancer immunotherapy, including chimeric antigen receptor T cells and bispecific T cell engagers, have improved outcomes, yet are limited by toxicity and immune escape by target downregulation. Thus, novel less toxic treatment modalities are highly warranted. The tumor mutational burden in ALL is low, which results in a low number of potentially immunogenic neo-antigens that could be used as targets for neo-epitope-specific therapeutic cancer vaccines. However, recent findings in solid cancer demonstrate that it is not the quantity but the quality of neo-antigens in the tumor that determine the tumor-specific immune response. Furthermore, novel sequencing techniques such as long-read sequencing and optical genome mapping can identify unknown genetic aberrations that may be targeted by neo-antigen vaccines. In ALL, both the *ETV6-RUNX1* and *BCR-ABL1* fusion genes, and the *RAS*-isoform mutations are frequent, and these genomic alterations generate immunogenic neo-epitopes. Additionally, therapeutic cancer vaccinations are well suited for ALL as the tumor burden is extremely low at time of a potential post-induction vaccination therapy, and patients are relatively young and are therefore less affected by immunosenescence. Thus, we envisage that neo-antigen specific therapeutic cancer vaccines could pose an important modality in future treatment algorithms for ALL.

## Introduction

Acute lymphoblastic leukemia (ALL) is an aggressive hematological cancer originating from immature B- or T-lymphoblasts primarily located in the bone marrow. Though rare, ALL is the most common pediatric malignancy. Mainstay treatment of ALL comprises a long course of intensive chemotherapy combined with high-dose corticosteroids. Though more than 90% of patients are cured, the therapeutic regimen has a treatment-related mortality of 4%, [1] and long-term side effects significantly impact patient quality of life. Hence, treatment modalities with lower toxicity are highly warranted.

The advent of cancer immunotherapy has changed the therapeutic landscape for many hematological cancers—especially for B-cell precursor-ALL (BCP-ALL) as both chimeric antigen receptor T cells (CAR-T) and bispecific T cell engagers (BiTE) targeting CD19 have become commercially available. [2, 3] Both CAR-T and BiTE however, have their limitations. For CAR-T, severe side effects such as the cytokine release syndrome (CRS) and Immune Effector Cell-Associated Neurotoxicity Syndrome (ICANS) are frequent, whereas these side effects are less severe for BiTE. [4] However, both treatments come at a great financial cost which limits the accessibility for a significant proportion of patients. As both CAR-T and BiTE demonstrate that the immune system can be used to cure ALL, other less intensive cancer immunotherapeutic treatment options such as therapeutic cancer vaccinations could potentially cure patients with ALL.

Given the advances in cancer immunotherapy for ALL outlined above, and recent advances of therapeutic cancer vaccines spurred by several recently published personalized neo-antigen vaccination trials, [5–7] we have reviewed current literature to explore the potentials of neo-antigen specific therapeutic cancer vaccines in ALL. First, we briefly describe ALL, immunotherapies for ALL, and therapeutic cancer vaccines and then describe the scientific evidence supporting the concept of neo-antigen specific cancer vaccines in ALL. Finally, we provide some thoughts on how these vaccines can be incorporated in existing and future treatment options for ALL.

### Acute lymphoblastic leukemia

ALL is the most common malignancy in childhood, with an age-adjusted incidence of 1.7/100.000 in the US. [8] The incidence peaks at age 2–5 and declines with age with 15% of patients being above 18 years. [1] The disease is characterized by infiltration of malignant, immature lymphoblasts in the bone marrow, blood, and extramedullary sites, including the central nervous system. BCP-ALL is the most frequent subtype, accounting for 85% of all cases. T cell (T)-ALL increases in frequency with age, accounting for 30% of adult cases, more often involves extramedullary sites, and has a lower overall survival rate (OS). In total, 10–13% of patients with ALL relapse, and these patients have a poor clinical outcome with a 5-year OS of < 50%. [1, 9]

ALL therapy comprises induction, consolidation, and maintenance therapy and includes high-dose glucocorticoids and combination chemotherapy. In addition, central nervous system prophylaxis, targeted therapies like tyrosine kinase inhibitors and immunotherapies, as well as allogeneic hematopoietic stem cell transplantation (allo-HSCT) for relapsed/refractory (r/r) ALL are used. [10] The prognosis for ALL has improved dramatically over the last 50 years, and 5-year OS is currently 94% for children (1–9 years), 87% for older children (10–17 years), and 78% for adults under 45 years of age. [1]

However, the high intensity therapeutic regimen has consequences. Overall treatment-related mortality in ALL is 3–5%, but considerably higher in certain subgroups. [1, 10] Furthermore, treatment-related toxicity is severe and affects a high proportion of patients, many of which have long-term consequences heavily affecting quality of life. Thus, considerable efforts to develop strategies for de-intensification and alternative, less toxic therapeutic approaches are ongoing. [1, 11]

### Immunotherapy for ALL, advances, and challenges

In ALL, a range of immunotherapies are currently being tested in clinical trials. [11, 12] For BCP-ALL, some of these have been implemented into current therapeutic regimens for r/r ALL. [11] These comprise inotuzumab ozogamicin (anti-CD22 monoclonal antibody (mAb) conjugated with calicheamicin), [13–15] anti-CD19 CAR-T, [3] and blinatumomab (CD19xCD3 BiTE), [2] which was recently approved for consolidation therapy in pediatric and adult patients with Philadelphia chromosome (Ph)-negative ALL. [16] For T-ALL, fewer therapies are available, but daratumumab (anti-CD38 mAb) and anti-CD7 CAR-T therapy have both shown promise for bridging to allo-HSCT in high-risk, relapsed disease. [17, 18]

Use of current immunotherapies in ALL comes with challenges. Both BiTE and CAR-T can cause toxicities associated with T cell activation, such as CRS and ICANS, both of which are most prevalent and severe in CAR-T treated patients. [19, 20] In T-ALL, an additional major challenge is identifying a suitable target, as tumor antigens shared between tumor cells and normal T cells can result in fratricide and subsequent severe T cell aplasia and immunodeficiency. A fundamental challenge for immunotherapies in both B- and T-ALL is relapse mediated by downregulation of target antigens by tumor cells. This is shown to be a major limitation to widespread use of CD19-directed therapies. [21] Hence, additional targets are needed to improve efficacy of cancer immunotherapy in ALL.

### Therapeutic cancer vaccines

The mechanism of action of therapeutic cancer vaccines relies on the ability of the vaccine to either induce or enhance the T cell response specific to one or several antigens expressed by tumor cells. [22] Tumor antigens are categorized as tumor-associated antigens (TAA) or tumor-specific antigens (TSA). The former comprises expression antigens, differentiation antigens or cancer–germline antigens. [23] TSA are generated due to various tumor-specific alterations such as genomic mutations, dysregulated RNA splicing and post-translational modifications and integration of viral open reading frames. [23, 24]

When designing a vaccine for therapeutic cancer vaccination, several aspects must be considered [25]: Survival of the cancer cell must be dependent on the expression of the antigen; epitopes derived from the antigen should be expressed stably as peptide:HLA complexes; and the patient needs to have T cells with high avidity T cell receptors for the peptide:HLA complex. This latter criterion is one of the reasons that neo-antigens are hypothesized to be superior targets for therapeutic cancer vaccines. Neo-antigen specific T cells are not deleted during the development of central tolerance thus, high-avidity neo-antigen specific T cells are more likely to emerge than high avidity TAA-specific T cells. The most well described source of neo-antigens are genomic mutations of which most frequently occurring are single nucleotide variations (SNV) such as the *RAS* and *TP53* mutations that generate immunogenic epitopes. [26, 27] Insertions and deletions (INDELs) are another common source of neo-antigens and though they are less frequent than SNV, they generate more immunogenic neo-antigens compared to SNV. An example of a shared INDEL which generates shared immunogenic neo-epitopes is the calreticulin exon 9 mutations. [28–30] Antigens formed by gene-fusions however, are believed to generate better neo-epitopes than both SNV and INDELs. An example is the BCR-ABL1 fusion protein in chronic myeloid leukemia (CML) and B-ALL. [31, 32] Apart from genomic mutations, transcriptomic variants may also generate neo-antigens. Transcriptomic variants arise due to alternative splicing, [33] altered post-translational modifications such as changes in glycosylation patterns, and translation of non-coding regions. [24] Though, transcriptomic variants are a potential rich source of neo-antigens, the full potential these targets are yet to be fully explored. Virally encoded antigens expressed by transformed cells are highly different compared to normal human proteins and hence, these proteins are a rich source of neo-antigens. [24]

Apart from choosing the right target for the vaccine another important aspect of vaccine design is the delivery platform and adjuvant. These should ensure a high level of antigen presentation by professional antigen-presenting cells (APC); a high level of co-stimulation delivered by APCs; a broad coverage of antigens presented by APCs; and a sustained presentation of antigen by APCs—all of which are critical in generating an effective immune response. [34] General challenges for the development of neo-antigen specific therapeutic cancer vaccines are the heterogeneous inter-patient mutational spectrum and the differences in HLA-types. As such, only highly frequent somatic mutations such as *KRAS*-mutations in solid cancer can be targeted by off-the-shelf therapeutic cancer vaccines [35, 36] which can be used for defined frequently occurring mutations in patients with a defined HLA-type. However, advances in genomic profiling and bioinformatic pipelines for neo-epitope predictions have ushered in a new era of semi-personalized therapeutic cancer vaccines targeting patient-specific neo-antigens. [37, 38]

### Genetic aberrations and somatic mutations in ALL

Many recurrent genomic aberrations can be identified in ALL including structural changes such as translocations, inversions, insertions, or deletions in addition to aneuploidy. Advances in genomic profiling techniques have broadened the genomic landscape of pediatric ALL significantly, and a distinct and likely founding event can now be characterized in more than 90% of patients, classifying them into one of > 20 subtypes. [39]

For BCP-ALL, these aberrations can be grouped according to clinical prognosis with a considerable variation of subtype distributions with age. Low-risk subtypes, such as *ETV6-RUNX1* and high hyperdiploid ALL are observed mostly in children, while high-risk subtypes such as *BCR-ABL1* ALL are seen mostly in adults. [1, 40] Likewise, mutations in the RAS pathway are highly frequent in high hyperdiploid cases. [41] *NRAS* mutations are the most prevalent of these and > 90% of these occur in exon 1 (codon 12/13). [41] Although considered cooperative rather than initiating, activating RAS pathway mutations are thought to play an important role in B-ALL leukemogenesis and progression, when present. [42] Unlike RAS pathway mutations, gene fusions such as *ETV6-RUNX1* and *KMT2A-AF4* are considered founding events in BCP-ALL. This is supported by studies of monozygotic twins with BCP-ALL, sharing the same fusion gene but harboring different secondary mutational events. These studies, among others, have led to the *two-hit hypothesis* regarding ALL leukemogenesis, where the initial first hit is thought to arise in utero, and the second genetic hit is thought to arise postnatally. [43] However, the molecular mechanisms of ALL are highly complex and variable and beyond the scope of this paper but has been extensively reviewed by Inaba and Mullighan. [44]

In T-ALL, genomic aberrations are not used for risk stratification. However, NOTCH1 pathway activating mutations are very common and have been associated with a good prognosis, while RAS/RAF/MEK/ERK deregulation delineates poorer outcomes. [39, 45] Gene fusions are also found in T-ALL. A large study of the T-ALL genomic landscape detected 83 chimeric in-frame fusions in 191 patients, of which the most frequently involved were *MLLT10*, *KMT2A*, *ABL1*, and *NUP98*. The same study found a mean of 15.8 mutations per case in the full cohort of 264 patients. [46] In an extended cohort of 2754 pediatric BCP- and T-ALL cases, the authors identified four putative somatic driver alterations per patient sample and a total of 376 potential driver genes. [41]

Recently, Brandes et al. used optical genome mapping (OGM) to detect several previously unidentified genomic aberrations in pediatric ALL and speculated that OGM in combination with RNA- and long-read sequencing can be used to ascertain the entire mutational spectrum in patients. [47] Hence, the range of genetic aberrations in ALL is likely to expand in the future.

### In silico* evidence of neo-epitopes in ALL*

Antigen immunogenicity relies both on stability of the antigen:HLA complex and on T cell receptor avidity to the antigen:HLA complex. Whereas antigen:HLA complex stability can sometimes be evaluated using in silico methods, detection of neo-antigen specific T cells requires functional in vitro assays. Chang and co-workers performed in silico analysis of 540 pediatric cancer genomes, of which 284 were from ALL patients, by whole-genome sequencing (WGS) and mRNA sequencing, with simultaneous analysis of patient HLA-I haplotypes and expression. [48] A median of six missense mutations and three 9-mer neo-epitopes in ALL were predicted. In 16 cases, ten or more potential 9-mer neo-epitopes were found. The highest average number of neo-epitopes was found in *ETV6-RUNX1*, hyperdiploid, and BCP-ALL without identified cytogenetic aberrations, while the lowest was found in infant ALL. Previously described neo-antigens formed by *RAS*-isoform mutations and the *BCR-ABL1* fusion protein were also predicted in this study, and the *KRAS* G13D mutation was found to generate a stable neo-antigen:HLA complex in 12% and 20% of hyperdiploid and hypodiploid B-ALL patients, respectively. Additionally, 21% of patients with Philadelphia chromosome (Ph)^+^-ALL were predicted to generate a stable neo-epitope:HLA complex formed by the *BCR-ABL1* fusion protein. Given the high frequency of the *ETV6-RUNX1* fusion in ALL, an interesting observation was the identification of *ETV6-RUNX1*-derived neo-epitopes in 68% of these patients. [48]

### In vitro* and *in vivo* evidence of neo-epitopes in ALL*

Though in silico prediction algorithms can be used to identify putative neo-epitopes, a high peptide:HLA affinity score does not necessarily confer a strong T cell response against the predicted neo-epitopes. The presence of T cells specific to the peptide:HLA complex and T cell mediated recognition of cells with endogenous expression of the target epitope is as prerequisite to establish a neo-epitope as a confirmed target. For B-ALL, the findings by Chang et al. were substantiated by neo-epitope prediction in nine patients (seven with *ETV6-RUNX1* and two with *DUX4-IGH*) based on tumor and germline whole-genome sequencing (WGS), whole-exome sequencing and tumor mRNA sequencing. [49] Somatic missense mutations and gene fusions were investigated and used to identify putative neo-epitopes restricted to patient-specific HLA alleles in all patients. An average of 14 binding neo-epitopes were found per patient. Most patients with identifiable *ETV6-RUNX1* genomic fusions, had neo-epitopes spanning the fusion point and ex vivo experiments on bone marrow derived CD8^+^ T cells demonstrated the ability of these cells to recognize *ETV6-RUNX1* derived peptides in seven of nine tested patients. Another important finding from this study was that several other somatic mutations were shown to generate immunogenic neo-epitopes, and that culturing patient CD8^+^ T cells with autologous tumor cells increased the frequency of neo-epitope specific T cells in vitro. [49] Similarly, a small-scale study in pediatric ALL showed that dendritic cells (DC) stimulated with predicted neo-epitopes induced a CD8^+^ T cell response against the predicted peptides in vitro. [50]

In addition to the identified neo-antigens mentioned above, a significant proportion of ALL patients have mutations that are known to generate well described neo-epitopes. *TP53* mutations are found in both newly diagnosed ALL and in relapsed pediatric B-ALL, and confer a poor outcome in both. [51, 52] Some of the *TP53* mutations in ALL generate both HLA-I and HLA-II epitopes. [26, 53] The *BCR-AB1L* fusion detected in both B-ALL and chronic myeloid leukemia (CML) generates fusion proteins p190, p210 b3a2 and p210 b2a2. Specific T cell responses to these have previously been described. [31, 54–56] Corroborating these results, a therapeutic cancer vaccination targeting the p210 fusion protein was tested in CML and shown to induce T cell responses to the vaccination epitope. [57] Encouragingly, one patient was ultimately cured after treatment with the vaccine [58], demonstrating the ability of neo-antigen specific T cell vaccines to induce a complete molecular response. Three patients with Ph^+^ ALL received adoptive T cell therapy with *BCR-ABL1* p190 specific T cells that were expanded in vitro. All patients attained a complete response after several T cell infusions demonstrating that *BCR-ABL1* directed immunotherapy is a potential feasible option in Ph^+^ ALL. [59] 

As recognized by Chang et al. and Li et al., a significant proportion of ALL patients have mutations in *NRAS* and *KRAS* [48, 60]*.* These mutations have received considerable attention as targets of neo-antigen-specific therapeutic cancer vaccines and were among the first somatic mutations to be targeted by such vaccines. [61] Several studies have described RAS neo-epitope-specific immune responses in both cancer patients and healthy donors, [62–65] and the induction of RAS-mutant specific immune responses by therapeutic cancer vaccines show evidence of a clinical effect. [35] Though RAS mutations are not founder mutations in ALL, they are highly prevalent in relapse, [41] rendering them attractive targets for relapse prevention in ALL. As both hypo- and hyper-diploid and Ph-like ALL lack proper neo-antigen targets in the form of fusion proteins but frequently harbor RAS-isoform mutations, targeting mutant RAS could be of relevance in these ALL subtypes. A recently published vaccination trial targeting mutant *KRAS* in solid cancer adds impetus to this notion. [66]

The studies above investigated the immunogenic potential of 9-mer neo-epitopes but not 8-, 10, and 11-mer epitopes, which are also presented by HLA-I. Furthermore, the majority of transformed cells in B-ALL express HLA-II [67], thereby rendering HLA-II-restricted neo-epitopes targeted by CD4^+^ T cells potential targets in B-ALL as well. Thus, the number of neo-epitopes in ALL is likely higher than demonstrated by the studies above. A clinical vaccination trial has been completed in patients with de novo or r/r ALL, utilizing peptide epitopes based on tumor exome and RNA sequencing data, as well as HLA-binding affinity predictions (NCT03559413). The vaccine was administered in combination with granulocyte–macrophage colony-stimulating factor (GM-CSF) and Imiquimod. No results have been reported yet.

### Tumor cell intrinsic challenges to neo-antigen specific vaccines in ALL

In theory, a main obstacle to therapeutic cancer vaccination in ALL is the low tumor mutational burden (TMB), which yields a low frequency of neo-antigens. This relationship was demonstrated in a study showing that TMB and number of generated neo-antigens is considerably lower in ALL compared to lung cancer and melanoma both of which have a high TMB. [48] However, the same study found that most of the analyzed cases of ALL form one or several neo-antigens. [48] These data are supported by in vitro data by Zamora et al. discussed above. [49] Targeting of several antigens is more beneficial per se, however targeting of a high number of neo-epitopes by vaccination might not be beneficial due to epitope immunodominance, [68] and as recently suggested neo-antigen quality is more important than quantity. [5, 69] Hence, the low number of neo-antigens formed in BCP-ALL might not be an issue for therapeutic cancer vaccines, if the neo-antigens formed are of sufficient quality to induce a robust immune response. A recent study in patients with renal cell carcinoma, which also has a relatively low TMB [70] substantiated this by demonstrating robust antigen- and tumor-specific immune responses in patients after vaccination with personalized neo-peptide vaccines. [71] Identification of genetic aberrations using novel methods such as OGM and long-read sequencing is also likely to yield more targets thereby expanding the possible neo-antigen repertoire. In another interesting study, DNA and RNA sequencing was used in combination with mass spectrometry proteomics to identify several immunogenic neo-epitopes in pediatric medulloblastoma—a cancer with a TMB comparable to ALL. [72]

Another challenge for the development of efficient therapeutic cancer vaccines in ALL and cancer in general is the intracellular proteasome-dependent processing of proteins leading to the generation of potentially antigenic peptides presented by HLA class I on the cell surface. [73] Although putative neo-epitopes around a fusion breakpoint can be shown to bind HLA class I molecules with high affinity and also to induce a T cell response in vitro, these will not be useful in a cancer vaccine if they are not endogenously produced by the transformed cells in vivo. In line with this, a study found that one of the immunodominant epitopes found in Zamora et al. was in fact not endogenously produced in a transgenic mouse model. [74] Zamora et al., however, demonstrated neo-epitope-specific CD8 T cell expansion after co-culture with autologous tumor cells for other putative neo-epitopes, indicating that these were indeed endogenously generated and presented. To overcome the challenge of proteasomal processing, in silico tools for post hoc filtering of putative neo-epitopes based on proteasomal cleavage prediction have been developed. [75]

The proteasome contributes in several ways to the expansion and complexity of the neo-epitope repertoire. First, the proteasome predominantly produces smaller peptides than the 8-11mer length fitting into the HLA class I groove, and studies suggest that combinations of two < 8mer peptides each binding one HLA- class I anchor residue, thus creating dual peptide occupancy, are in fact able to elicit a combinatorial epitope-specific CD8 T cell response. [76] In addition, some studies show that the proteasome can re-ligate peptides and hereby generate spliced epitopes, comprising a varying but significant part of the immunopeptidome. [77, 78] Thus, spliced epitopes could potentially expand the pool of putative cancer vaccine targets, although conflicting results regarding the benefit of this approach have been published. [73, 77]

### Tumor cell extrinsic challenges to neo-antigen specific vaccines in ALL

Apart from the low TMB, a substantial hurdle to therapeutic cancer vaccination for both B-ALL and T-ALL is that the immune system itself is affected by the disease both quantitatively and qualitatively. Th1 cells are decreased in the bone marrow at diagnosis of B-ALL [79], and chemotherapy influences the proliferative capacity of both naïve and antigen experienced effector T cells [80, 81], which may impact how well the adaptive immune system is primed by the vaccines. In the setting of allo-HSCT, this question remains even more complicated, as full T cell reconstitution is only achieved after 6–12 months, [82] thereby leaving the optimal timing for administration of therapeutic cancer vaccines unresolved.

The tumor microenvironment (TME) in T-ALL is immunosuppressive with an overexpression of the immunosuppressive proteins such as galectin-9 which inhibits the tumor-specific immune response. In B-ALL higher frequencies of non-classical monocytes have been identified in the bone marrow of patients at relapse. [79] Myeloid cells are known suppressors of the tumor-specific immune response; [83] however, vaccination against immunosuppressive cells such as myeloid cells was suggested as a method to counter the immune suppression induced by immunoregulatory cells in the TME. [84–86]

### Opportunities of neo-antigen specific vaccines in ALL

Though some challenges need to be solved before neo-antigen-specific vaccines can be used for ALL, several disease-specific features of ALL may render it suitable for therapeutic cancer vaccines. Firstly, the relatively young age of patients with ALL will most likely have a positive impact on the efficacy of therapeutic cancer vaccination as young individuals are less immunosenescent with a relatively high fraction of naïve T cells and a high replicate capacity of effector immune cells. [87] Secondly, in most patients, induction therapy decreases the bone marrow tumor burden to very low levels—minimal residual disease (MRD) corresponding to less than 1 leukemic cell per 10,000 bone marrow-derived hematopoietic cells. [88, 89] Several trials of cancer vaccination have shown the tumor burden to be of significance to the clinical response. [7, 90] One likely explanation is that effector T cells induced by the vaccine are a finite resource since a limited amount of T cells—the tumor-specific T cells—are available to kill the transformed cells. T cells are not able to kill an indefinite number of target cells and require time to detect, engage and kill their target. [91] If the transformed cells outnumber the effector T cells, the latter are outpaced by the rapidly proliferating transformed cells thereby resulting in a relapse. This has been contemplated to be one of the mechanisms behind failure of immune checkpoint inhibitors in ALL. [49] Furthermore, this effector-cell:tumor-cell imbalance was provided as an explanation to the failure of a neo-antigen-specific cancer vaccine in patients with chronic myeloproliferative neoplasms, which are hematological cancers with a very high tumor burden. [92] The sensitive and feasible quantification of tumor burden by MRD monitoring allows for an individualized MRD-guided vaccine initiation timeframe, which may also allow for later inclusion of high-risk patients that do not initially reach undetectable MRD status.

**Fig. 1:**
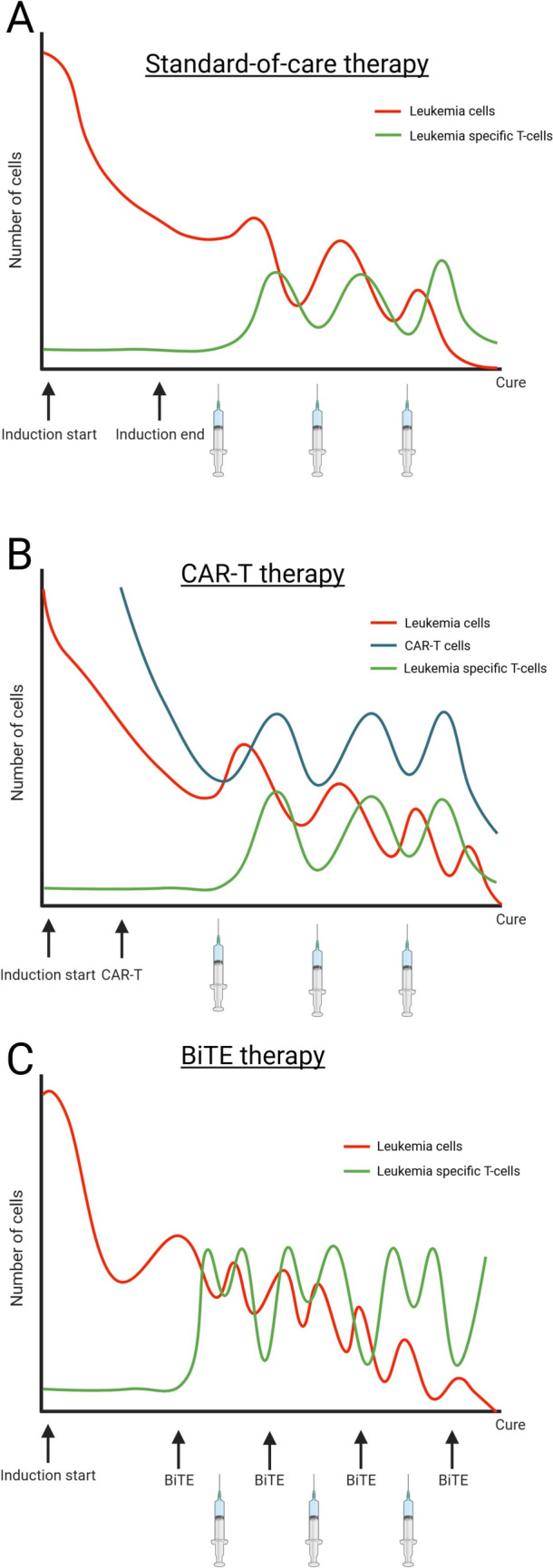
Timeline depicting how the number of tumor cells and effector immune cells may be affected by therapeutic cancer vaccines in combination with different treatment modalities for ALL. **A** Tumor cell load and number of effector T cells (*y*-axis) during a standard-of-care first-line treatment regimen for ALL. The burden of leukemia cells (red line) is significantly reduced during induction therapy. The frequency of leukemia-specific T cell (green line) is increased by vaccines. The tumor burden gradually declines and disappears over time as the leukemia-specific T cells are sustained by repeated vaccines. **B** Tumor cell load (red line), number of CAR-T cells (blue line), and leukemia specific T cells (green line) during a treatment schedule with CAR-T after induction therapy. The tumor burden is vastly reduced by induction therapy, and the CAR-T decreases the burden even more. Therapeutic cancer vaccines are administered repeatedly to sustain the frequency of CAR-T cells and leukemia-specific T cells which ultimately clear the residual tumor cells. **C** Tumor burden (red line) and number of leukemia specific T cells (green line) during consolidation therapy with BiTE. The tumor burden is reduced by induction therapy. BiTE induces a strong leukemia-specific T cell response that is enhanced by therapeutic cancer vaccines. BiTE and vaccines could be used simultaneously or interchangeably (as depicted in this figure). Figure created with biorender.com

The failure of CAR-T and BiTE to demonstrate convincing clinical responses in solid cancers is partly ascribed to the inability of T cells to effectively penetrate the tumor microenvironment, which is often fibrotic and poorly vascularized. [93] In ALL, high response rates to CAR-T and BiTE is believed to be partly conferred by the ability of tumor-specific T cells to easily infiltrate the tumor microenvironment—the bone marrow—which would likewise be easily accessible for vaccination-induced effector T cells. As CAR-T and BiTE are used increasingly for treatment of B-ALL, and combination of CAR-T and vaccination has been demonstrated to have an effect in B-ALL [94], one opportunity may be to apply neo-antigen therapeutic cancer vaccines as maintenance therapy following treatment with CAR-T or BiTE. Another possibility is administering vaccinations between BiTE treatment cycles (Fig. 1).

### Combining neo-antigen-specific vaccines with other treatments

The theoretical rationale behind combining vaccines with BiTE and CAR-T is the concept of epitope spreading. [95] As CAR-T and BiTE facilitate immune-mediated killing of malignant cells, TAA and TSA are shed by dying tumor cells, and are phagocytosed, processed and presented by DC. As the malignant cells are cleared in a highly inflammatory microenvironment, the DC will potentially prime TAA- and TSA-specific T cells. The effector functions of these T cells will be supported by therapeutic cancer vaccination. As vaccination after BiTE or CAR-T could potentially enhance the T cell response to non-CD19 tumor antigens, vaccines can theoretically decrease the rate of CD19^neg^ relapses—a common cause of relapse in BiTE or CAR-T treated B-ALL. [21] Though the concept of combining CAR-T and therapeutic DC cancer vaccines using have been demonstrated in a clinical trial [94], more evidence is needed to support this idea.

Cytotoxic agents and ionizing radiation induce immunogenic cell death (ICD) —a process that increases the immune response to antigens expressed by cells undergoing ICD. [97] In acute myeloid leukemia, patients with increased expression of ICD markers displayed a more pro-inflammatory immune phenotype and gene expression profile, enhanced immune responses to TAA, and improved overall survival. This suggests that ICD generates an AML-specific immune response which translates to improved survival. [98] Though, such a mechanism would need to be investigated closer in ALL, we hypothesize that cytotoxic agents used for treatment of ALL could induce ICD and thereby an ALL-specific immune response, which can potentially be enhanced by therapeutic cancer vaccination.

### Future directions

In light of the overwhelming success of BiTE and CAR-T for BCP-ALL, it might seem unnecessary to provide another cancer immune therapeutic modality for this disease. However, current treatments are associated with severe toxicity and not all patients are cured, thus further work is needed for both de novo and r/r ALL. To that end, therapeutic cancer vaccines are easily combined with existing therapies and have very few side effects. [96] Though CAR-T and BiTE are highly beneficial and likely to move into the first-line treatment of BCP-ALL based on recent promising studies, [16] it is important to consider that both treatments are costly and not readily available at every center of referral. As standard induction and consolidation therapies induce deep remissions in most patients, therapeutic cancer vaccines could be ideal to administer after consolidation therapy as the tumor burden at this time-point is low and the vaccine-induced neo-antigen-specific immune response may clear the residual transformed cells. Therefore, we hypothesize that patients with ALL may benefit from therapeutic cancer vaccination.

A major challenge is to identify suitable targets for therapeutic cancer vaccination. Wilms tumor antigen-1 has shown clinical potential as a vaccination target in acute myeloid leukemia. [99, 100] However, this antigen is not overexpressed in BCP-ALL, [101] rendering it a less attractive target. The cancer–germline antigen termed preferentially expressed antigen of melanoma (PRAME) is overexpressed in B-ALL, and peripheral blood T cells from ALL-patients display strong responses to PRAME derived epitopes. [102, 103] Another study demonstrated generation of T cell lines specific to several TAA such as PRAME, survivin and melanoma associated antigen (MAGE) [104], and Tyner et al. demonstrated enhanced expression of survivin by lymphoblasts in ALL and that survival of these was dependent on the expression of survivin. [105] In another study, MAGE was overexpressed by lymphoblasts. [106] Given the immunogenic potential of both survivin, MAGE and PRAME, these TAA could be potential targets for therapeutic cancer vaccines in ALL. Yet, none of these have been tested in clinical vaccination trials in ALL, and given the failure of any of these vaccine targets to enter the clinic in both solid and hematological cancer, neo-antigens should be investigated as targets for therapeutic vaccines in ALL.

**Fig. 2:**
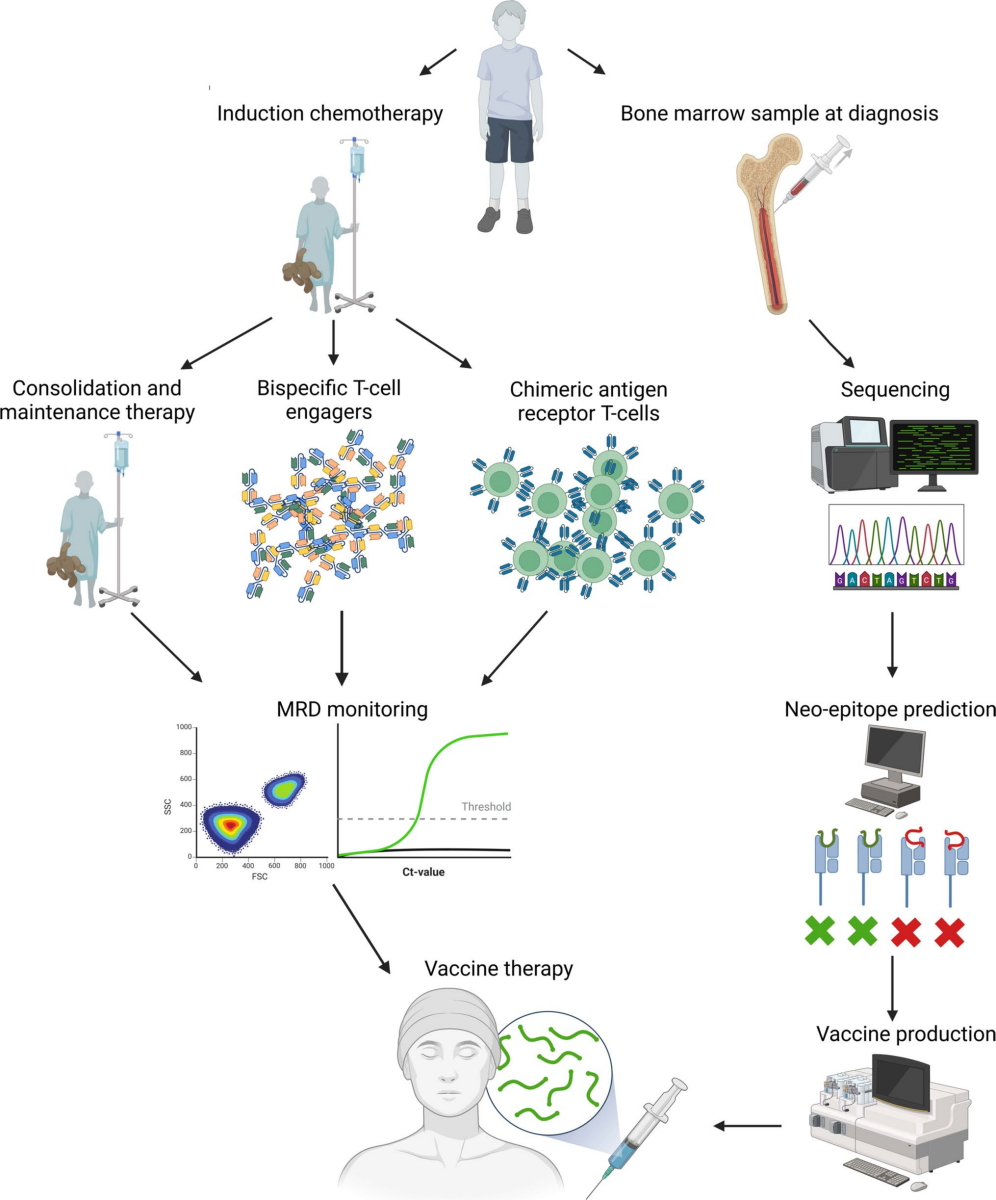
Workflow of the development of a personalized neo-antigen specific vaccine in ALL in conjunction with either standard therapy or cancer immunotherapeutic modalities. The vaccine will be produced as the patient undergoes induction chemotherapy and subsequent standard consolidation or consolidation with bispecific T cell engagers or chimeric antigen-receptor T cells. During standard consolidation therapy patients will be monitored for minimal residual disease (MRD) using standardized fluorescence activated cells sorting and quantitative PCR. After consolidation therapy, vaccines will be administered. Figure created with biorender.com

The low TMB resulting in a low number of immunogenic epitopes is a challenge that needs to be addressed and a readily available off-the-shelf therapeutic cancer vaccination for ALL is unlikely to be developed. However, lessons learned from therapeutic cancer vaccination trials with personalized vaccines in other cancers could be adopted. [5, 68, 71, 107] Several mutations in ALL generate immunogenic neo-antigens that are expressed by the transformed cells, and therapeutic cancer vaccines targeting these neo-epitopes can possibly have a clinical effect. The epitopes formed are mostly unique to each patient, hence to provide a neo-antigen-specific cancer vaccine one would need to adopt the principle of designing personalized therapeutic cancer vaccines. This is not trivial, as sequencing of the tumor sample is required, along with a bioinformatics pipeline to detect mutations that are most likely to generate immunogenic neo-epitopes and finally a manufacturing facility to produce the vaccine in a timely manner (Fig. 2). [38] This time-consuming workflow is a major hurdle as ALL is a disease with a high proliferation rate of the transformed cells. Thus, the disease may relapse quickly and in a short time manifest with a relatively high tumor burden which vaccine-induced T cells are unable to clear. Another challenge lies in regard to the targeted epitopes. Due to time and tissue constraints, epitope predictions would most likely need to be performed on diagnostic tumor tissue. Because of high selection pressure on transformed cells during induction therapy, one cannot be sure that neo-epitopes selected through analysis of the diagnostic sample are expressed by residual cancer cells after induction therapy. ALL displays clonal evolution from diagnosis to relapse [108] and it is thus of importance to choose targets that are not lost due to selection pressure. For instance, although RAS mutations generally are enriched at time of relapse, [108] Wanders et al. showed that a third of examined patients with RAS pathway mutations at diagnosis displayed multiple subclonal mutations, which in the majority of cases converged to one or two during relapse. [109] This underpins the importance of targeting founding mutations such as gene fusions for therapeutic vaccines. However, the immunogenicity of gene fusion products is not guaranteed.

## Conclusion

ALL is the most common type of pediatric cancer, and though most patients are cured, standard of care therapy confers a high rate of serious long term side effects. Emergence of cancer immunotherapy such as CAR-T and BiTE has improved the prognosis for r/r BCP-ALL considerably, however given the elaborate treatment process and high cost, new modalities are warranted. Several in vitro and in silico studies show that ALL generates immunogenic neo-epitopes, and the advent of personalized peptide and mRNA vaccines incorporating advanced bioinformatic algorithms for optimal epitope selection offers a promising solution to epitope selection and thus a possibility to bring therapeutic cancer vaccines for ALL into the clinic. Furthermore, novel, more sensitive methods for genomic sequencing may be used to find previously unidentified mutational targets. Another challenge will be to decide on when to start the therapeutic vaccines as the intensive standard of care therapy substantially impacts the composition and functionality of the cellular immune system. Since therapeutic cancer vaccines are generally safe and only rarely confer serious adverse events, we envisage that vaccines can easily be incorporated into current and future treatment algorithms. Epitope spreading may enhance the therapeutic effect of cancer immunotherapy such as CAR-T and BiTE and thereby potentially decrease morbidity and mortality in patients with ALL.
